# Novel *SEPN1* Mutations in Exon 1 Are Common in Rigid Spine With Muscular Dystrophy Type 1 in Chinese Patients

**DOI:** 10.3389/fgene.2022.825793

**Published:** 2022-03-16

**Authors:** Yanbin Fan, Zhifei Xu, Xing Li, Feng Gao, Enyu Guo, Xingzhi Chang, Cuijie Wei, Cheng Zhang, Qing Yu, Chengli Que, Jiangxi Xiao, Chuanzhu Yan, Zhaoxia Wang, Yun Yuan, Hui Xiong

**Affiliations:** ^1^ Department of Pediatrics, Peking University First Hospital, Beijing, China; ^2^ Department of General Pediatrics, Beijing Children’s Hospital, Capital Medical University, Beijing, China; ^3^ Department of Neurology, Children’s Hospital, Zhejiang University School of Medicine, Hangzhou, China; ^4^ Department of Pediatrics, Jining No. 1 People’s Hospital, Jining, China; ^5^ Department of Respiratory and Critical Care Medicine, Peking University First Hospital, Beijing, China; ^6^ Department of Radiology, Peking University First Hospital, Beijing, China; ^7^ Department of Neurology, Qilu Hospital, Shandong University, Qingdao, China; ^8^ Department of Neurology, Peking University First Hospital, Beijing, China

**Keywords:** congenital muscular dystrophy with spinal rigidity, rigid spine with muscular dystrophy type 1, respiratory insufficiency, *SEPN1* gene, selenoprotein N

## Abstract

Congenital muscular dystrophy with early rigid spine, also known as the rigid spine with muscular dystrophy type 1 (RSMD1), is caused by *SEPN1* mutation. We investigated the clinical manifestations, pathological features, and genetic characteristics of 8 Chinese RSMD1 patients in order to improve diagnosis and management of the disease. Eight patients presented with delayed motor development, muscle weakness, hypotonia, and a myopathic face with high palatine arches. All patients could walk independently, though with poor running and jumping, and most had a rigid spine, lordosis, or scoliosis. The symptoms of respiratory involvement were present early, and upper respiratory tract infections and pneumonia often occurred. Five patients had severe pneumonia, pulmonary hypertension, and respiratory failure. Lung function tests showed variable restrictive ventilation dysfunction. Polysomnography suggested hypoxia and hypoventilation. The serum creatine kinase (CK) level was normal or mildly increased. Muscle biopsy indicated chronic myopathic changes and minicores. Muscle magnetic resonance imaging (MRI) showed diffuse fatty infiltration of the gluteus maximus and thigh muscle. *SEPN1* gene analysis revealed 16 compound heterozygous variants, 81.3% of which are unreported, including 7 exon 1 variants. Our study expands the spectrum of clinical and genetic findings in RSMD1 to improve diagnosis, management, and standards of care. *SEPN1* mutations in exon 1 are common and easily missed, and exon 1 should be carefully analyzed when RSMD1 is suspected, which will provide valuable genetic counseling for the family and useful information for future natural history studies and clinical trials.

## Introduction

Congenital muscular dystrophy with early rigid spine, also known as the rigid spine with muscular dystrophy type 1 (RSMD1), is caused by *SEPN1/SELENON* (MIM 606210) mutation. RSMD1 is characterized by slowly progressive muscle weakness, early-onset spinal rigidity, scoliosis, and progressive and potentially life-threatening respiratory insufficiency induced by diaphragm weakness (Koul et al., 2014).

In addition to RSMD1, *SEPN1* gene mutations also cause congenital fiber-type disproportion, a severe form of classic multiminicore myopathy, and desmin-related myopathy with Mallory body-like inclusions (Cagliani et al., 2011). Due to the clinical and genetic overlap between these four autosomal recessive conditions, there is an emerging idea that they are all manifestations of the same disease, namely, *SEPN1*-related myopathy (*SEPN1*-RM) (Scoto et al., 2011). However, there is no clear correlation between genotype and pathologic or genetic findings.

The *SEPN1* gene, located on chromosome 1p36.13, contains 13 exons and encodes selenoprotein N, encompassing transmembrane domain, EF-hand domain (Ca^2+^-interacting region), thioredoxin domain, and SCUG motif (Chernorudskiy et al., 2020; Zhu et al., 2021). The underlying mechanisms of *SEPN1*-RM are unclear as the function of the selenoprotein N is incompletely understood. Selenoprotein N, an endoplasmic reticulum (ER) glycoprotein and an enzyme containing a selenium atom, has an established role in redox-based calcium hemostasis and protects muscle cells from oxidative damage and ER stress (Arbogast et al., 2009; Arbogast and Ferreiro, 2010; Pozzer et al., 2019; Varone et al., 2019). Mutations in *SEPN1* lead to the absence or malfunction of selenoprotein N, and one study has suggested that some compounds effectively rescued the *SEPN1*-devoid cell phenotype *ex vivo* (Arbogast et al., 2009). However, the lack of natural history data, the complications of disease progression or prognosis determinants, and unclear phenotype–genotype correlations hinder the clinical trial implementation.

In this study, we investigated the clinical manifestations, pathological features, and genetic characteristics of 8 Chinese RSMD1 patients in order to improve the diagnosis and management of this disease.

## Materials and Methods

### Clinical Studies

We studied eight patients with a clinical diagnosis of *SEPN1*-RM, performed a retrospective review of medical records, and carried out a prospective cohort study. Clinical and laboratory data, including motor development, family history, serum creatine kinase (CK) levels, pulmonary function test and polysomnography data, electrocardiography (ECG), ultrasound cardiography, and electromyography (EMG) findings, and muscle magnetic resonance imaging (MRI) were collected and analyzed based on the medical records provided by the attending physicians.

### Pathological Studies

Skeletal muscle biopsies were performed on probands for pathological diagnosis. Muscle biopsies from the biceps brachii or quadriceps muscle were embedded, frozen in isopentane, and cooled in liquid nitrogen. Frozen sections (6 μm) were cut and processed for routine historical and immunohistochemical staining. A Nikon Eclipse TE2000-S microscope was used to obtain images.

### Genetic Analysis

Genomic DNA was extracted from peripheral blood leukocytes using standard protocols. The genomic DNA was first prepared as a DNA library, amplified, and then analyzed by next-generation sequencing (NGS) using a myopathy panel or whole-exon sequencing. After sequencing, the sequencing reads were mapped to the GRC37/hg19 human reference genome using Burrows–Wheeler Aligner software (version 0.59). Then the single-nucleotide variants (SNVs) and small insertions or deletions (InDels) were detected using the GATK UnifiedGenotyper (broadinstitute.org/). Finally, we filtered and selected the variants according to the minor allele frequency (MAF) with a cutoff value of <0.05 in multiple databases (such as 1000 Genome Project, dbSNP, HGMD, HapMap, and in-house Chinese local database). Variants’ pathogenicity was evaluated using the variant-classification guidelines of the American College of Medical Genetics and Genomics (ACMG) (Richards et al., 2015) and determined based on the population frequency, phylogenetic conservation analysis, *in silico* prediction programs, and reporting in locus-specific databases. The candidate variants detected using NGS were verified in the probands and family members using Sanger sequencing. Exon 1 of *SEPN1* and its flanking intronic regions were amplified by polymerase chain reaction (PCR) and directly sequenced using Sanger sequencing. The primers of exon 1 are as follows: F- 5′-CTT​GGC​GTT​CCG​GTG​TAG-3′, R- 5′-ACT​CGT​CCA​TGC​CCA​TGT​C-3′; F- 5′-GAT​CAG​CCC​CTC​TTG​GCG​TTC-3′, R: CTC​TGG​ATA​GAG​ACC​CCT​CCA​GTC-3′. The reference sequence NM_020451 of *SEPN1* was used.

## Results

In total, eight patients were clinically diagnosed with RSMD1, and *SEPN1* gene mutations were confirmed. The clinical features of these patients are summarized in Tables 1, 2.

**TABLE 1:** Clinical features of 8 RSMD1 patients.

Patient	Sex/age	Age of onset	Onset of symptoms	Maximum motor milestone	Contractures	Spinal deformities	CK level (U/L)	ECG	UCG	EMG	Muscle biopsy
1	F/9 y 3 m	Birth	Delayed motor milestones and weakness	Walking and running	Long finger flexor	Rigid spine and mild scoliosis	281	Normal (9 y)	Normal (9 y)	Myogenic	No significant pathological changes (1 y 2 m)
2	M/18 y	Birth	Delayed motor milestones and weakness	Walking	No	Rigid spine and scoliosis	<195	Sinus tachycardia (12 y)	Right ventricular enlargement, interventricular septal hypertrophy, pulmonary hypertension and mild tricuspid regurgitation (12 y); normal (18 y)	Myogenic	Myopathic, minicore (12 y)
3	F/11 y 9 m	<6 m	Delayed motor milestones	Walking	No	Rigid spine, scoliosis, and lordosis	<195	n/a	Mild enlargement of the right heart and mild pulmonary hypertension (6 y 8 m); normal (11 y)	No significant changes	n/a
4	F/12 y 4 m	Birth	Delayed motor milestones and weakness	Walking	No	Rigid spine and scoliosis	<195	Sinus tachycardia (8 y)	Normal (12 y)	No significant changes	n/a
5	F/8 y deceased	Birth	Delayed motor milestones and weakness	Walking and running	No	Rigid spine	<195	Sinus tachycardia (5 y)	Normal (8 y)	No significant changes	Myopathic, minicore (3 y)
6	M/13 y 8 m	1 y	Frequent falls and difficulty running	Walking and running	Elbow, Achilles tendon	Rigid spine, and mild scoliosis	664	n/a	Normal (13 y)	Myogenic	Myopathic, minicore (3 y)
7	F/13 y 5 m	<1 y	Delayed motor milestones and difficulty jumping	Walking	No	Scoliosis	<195	Sinus tachycardia (12 y)	Enlargement of right heart, decreased diastolic function of left ventricle, mild pulmonary hypertension, and mild tricuspid regurgitation (12 y)	Myogenic	n/a
8	F/7 y	<1 y	Delayed motor milestones	Walking, running	No	No	<195	Normal (7 y)	Normal (7 y)	n/a	n/a

F, female; M, male; y, year; m, month; CK, creatine kinase; ECG, electrocardiogram; UCG, ultrasound cardiography; EMG, electromyography; n/a, not available. The upper limit of the normal CK level is 195 U/L.

**TABLE 2:** Respiratory function of 8 *RSMD1* patients.

Patient	Lung function tests		Polysomnography						Severe pneumonia/Age	Respiratory insufficiency/Age	NIV/age
	Age	FVC (% predicted)	Age	Mean nocturnal SpO2 (%)	Minimum nocturnal SpO2 (%)	NH	AHI (/h)	OSAHS			
1	6 y 4 m	75.5	6 y 4 m	96	76	Yes	11.2	No	No	No	No
2	12 y	50.0	12 y	93	60	Yes	n/a	No	No	Yes/12 y	Yes/12 y
			12 y (NIV)	95 (NIV)	80 (NIV)						
3	8 y 7 m	32.2	6 y 3 m	77	23	Yes	41.5	Yes	Yes/5 y	Yes/5 y	Yes/6 y
			9 y (NIV)	97 (NIV)	93 (NIV)						
4	10 y 11 m	31.3	7 y 2 m	91	43	Yes	23.7	Yes	Yes/7 y	Yes/7 y	Yes/7 y
			10 y (NIV)	94 (NIV)	44 (NIV)						
5	6 y 3 m	33.7	6 y 3 m	88	64	Yes	15	No	Yes/5 y	Yes/5 y	Yes/5 y
6	12 y 11 m	42.8	12 y 11 m	82	60	Yes	47.3	Yes	No	No	Yes/13 y
			13 y (NIV)	96 (NIV)	93 (NIV)						
7	12 y 7 m	36.6	12 y	90	80	Yes	n/a	No	No	Yes/12 y	Yes/12 y
			12 y (NIV)	98 (NIV)	93 (NIV)						
8	7 y	n/a	n/a	n/a	n/a	No	n/a	No	No	No	No

y, year; m, month; FVC, forced vital capacity; NH, nocturnal hypoventilation; AHI, apnea–hypopnea index; h, hour; OSAHS, obstructive sleep apnea–hypopnea syndrome; NIV, noninvasive ventilation; n/a, not available.

### Clinical Characteristics

#### Onset of Signs

The age of onset ranged from birth to 1 year old. Five patients (5/8, 62.5%) had a congenital presentation with hypotonia (5/5) and feeding difficulties (3/5). All seven patients presented with delayed motor milestones or gross motor difficulties, subdivided into the following categories: 5/8 (62.5%) poor or delayed head control (after 4 months of age), 4/8 (50.0%) delayed ability to sit unsupported (after 10 months of age), 4/8 (50.0%) delayed walking (after 18 months of age), and 8/8 (100%) difficulty in running or jumping, frequent falls, or easy fatigability.

#### Muscle Weakness and Functional Abilities

Four patients (4/8, 50.0%) presented with facial weakness, high palatine arches, and a nasal, high-pitched voice. All patients had muscle weakness, especially axial weakness, in the neck flexors, abdominal muscles, and predominantly mild proximal limb muscles, with relatively well-preserved neck extensors. Deep-tendon reflexes were invariably diminished or absent. All patients remained fully ambulant and were able to walk independently indoors and outdoors when last followed up.

#### Spine and Joint Deformity

Six patients (except patients 7 and 8) were first referred for rigid spines between 4 and 12 years old (mean age 6.8 years). In 6 patients, presentation with scoliosis was between 4 and 12 years of age (mean age 8.8 years); patient 3 presented lordosis at 6 years of age. Patient 6 showed contractures involving the elbow and Achilles tendon at 12 years old. After birth, the right ring finger, left pinky finger, and ring finger of patient 1 could not be straightened, and the contracture of the long finger flexor was more pronounced at the age of 1 year old.

#### Respiratory and Cardiac Involvement

The symptoms of respiratory involvement were present in all patients. They often had upper respiratory tract infections, five (5/8, 62.5%) were susceptible to pneumonia, and three (3/8, 37.5%) had severe pneumonia between the ages of 5 and 7 years.

The respiratory function data of 8 patients are summarized in Table 2. Forced vital capacity (FVC) data were available, and all had predicted FVC values (FVC%) below 80%. Pulmonary function tests suggested mild (in patient 1), moderate (in patients 2 and 6), and severe (patients 3, 4, 5, and 7) restrictive ventilation dysfunction. Polysomnography indicated variable hypoxia and nocturnal hypoventilation in all patients (see Table 2), and three (3/8) showed low oxygen saturation, apnea, and a high apnea–hypopnea index (AHI), suggestive of obstructive sleep apnea–hypopnea syndrome (OSAHS). Five patients had varying degrees of respiratory insufficiency from 5 to 12 years old (mean age 8.2 years; median 7), requiring nocturnal noninvasive ventilation (NIV) at a mean age of 9.2 years (median 9.5). After NIV treatment, all patients slept well at night, the symptoms of hypoxia and hypoventilation improved, and oxygen saturation at night improved significantly compared with before. Patient 6 declined regular respiratory follow-up and nocturnal NIV, and died at 8 years old. Patients 1 and 8 did not receive NIV in the first decade.

All patients underwent cardiac assessment. Three (3/8) had sinus tachycardia without abnormal electrocardiogram. Although right ventricular enlargement and mild pulmonary hypertension secondary to respiratory insufficiency were observed in the echocardiogram of three patients (3/8, 37.5%), their cardiac function improved after the active non-invasive respiratory support. A normal echocardiogram was obtained for the remaining 5 patients.

#### Survival

Patient 6 declined regular nocturnal NIV and died from respiratory failure at the age of 8 years because of severe pneumonia.

#### Auxiliary Tests

Serum CK levels were normal or mildly increased in all patients (see Table 1). EMG revealed myogenic changes in 4/8 patients, and no significant changes were found in 3/8 patients.

#### Muscle MRI

**FIGURE 1:**
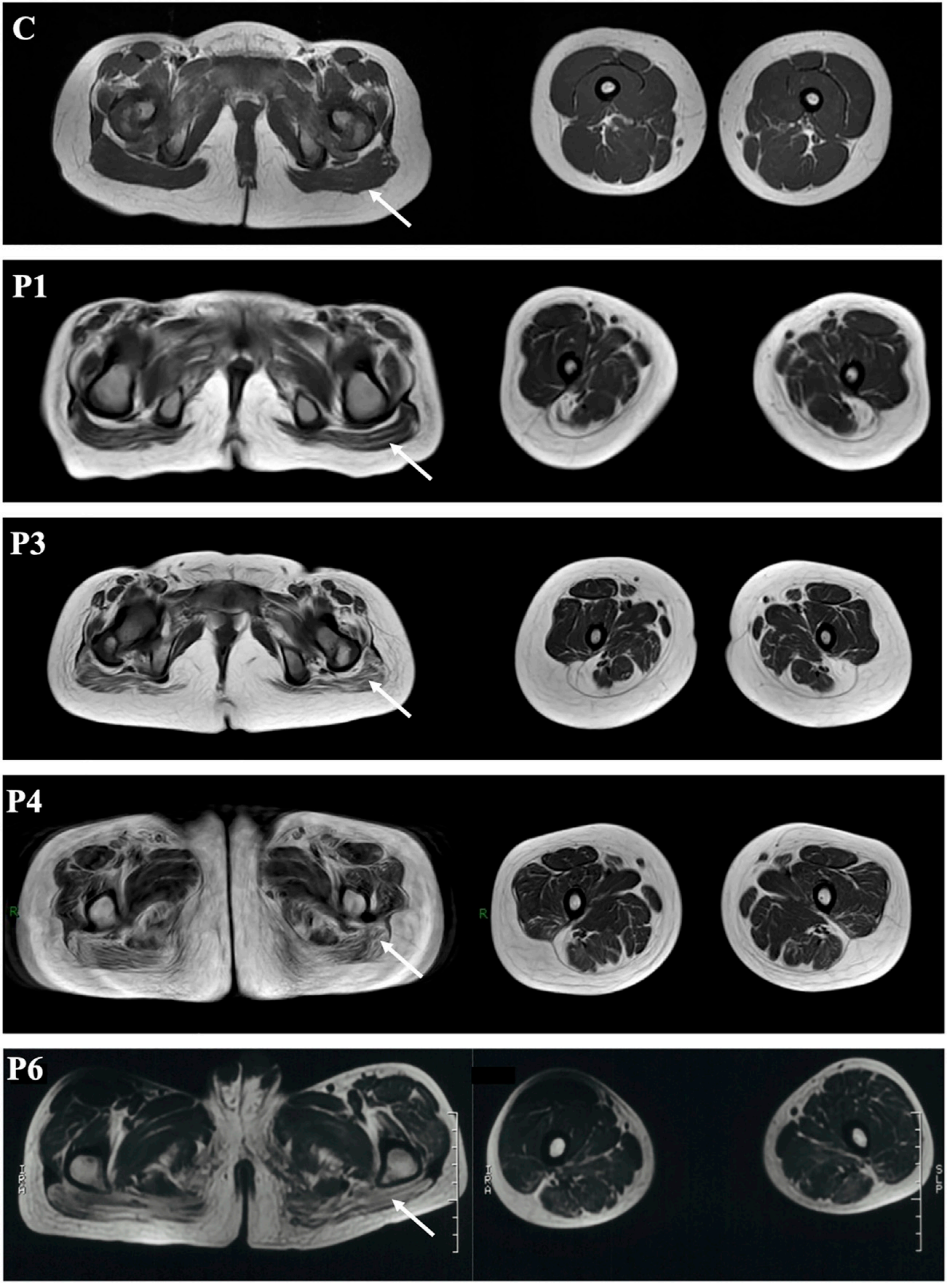
Muscle MRI of RSMD1 patients. Arrows showed the gluteus maximus and thigh. C: control.

Muscle MRI of probands 1, 3, 4, and 6 showed diffuse fatty infiltration of the gluteus maximus and thigh muscle (Figure 1). All the four patients exhibited fatty infiltration and atrophy of the gluteus maximus, and the adductor magnus was severely affected in patients 4 and 6. The semimembranosus was severely atrophic and absent in patient 1. Several muscles were preserved and mildly affected, including the gracilis, semitendinosus, rectus femoris and tibialis anterior, and tibialis posterior muscles in the lower leg (patients 1 and 6).

#### Muscle Pathology

**FIGURE 2:**
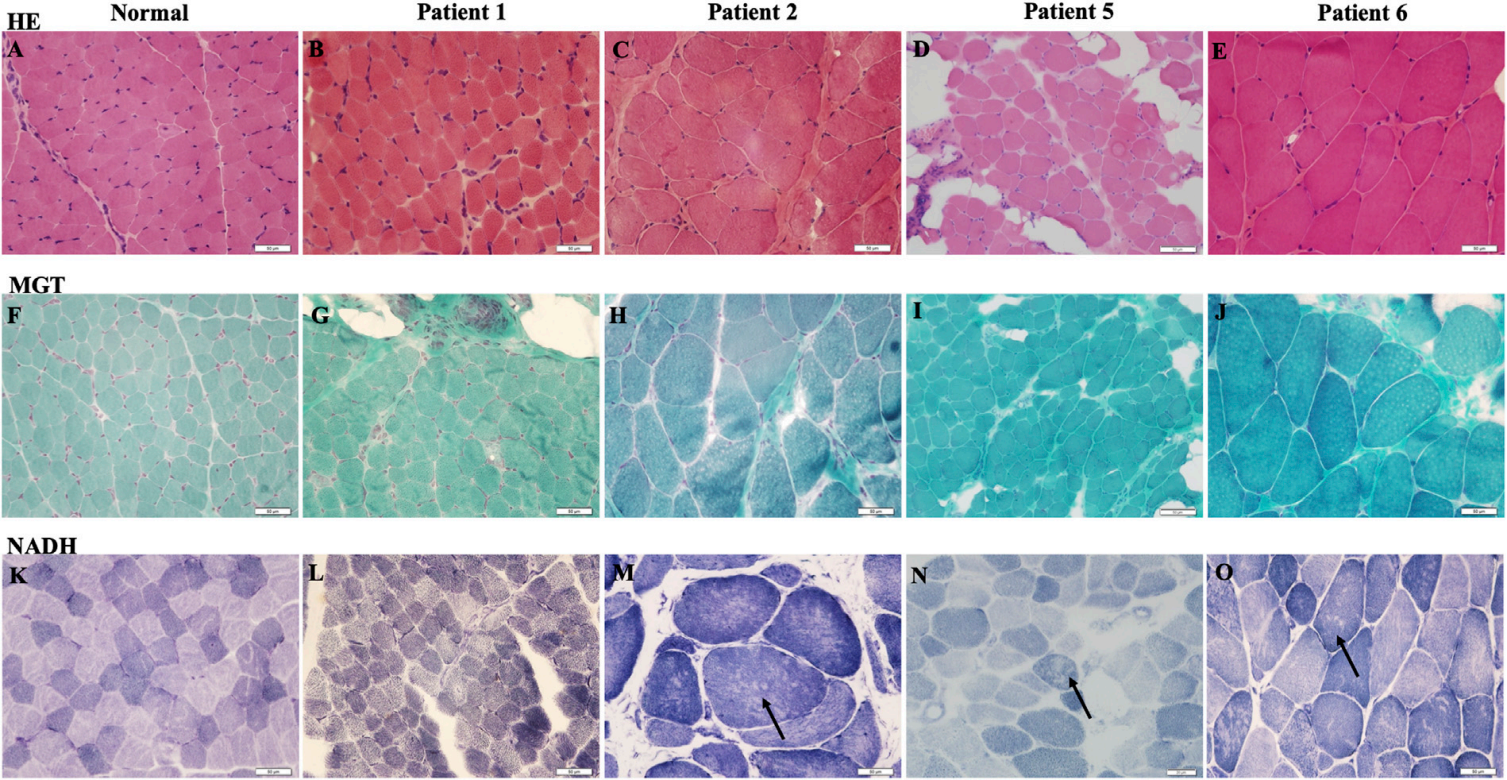
Muscle biopsy of RSMD1 patients: **(A–E)** hematoxylin and eosin (H&E) staining, **(F–J)** modified Gomori Trichrome (MGT) staining, and **(K–O)** β-nicotinamide–adenine dinucleotide (NADH) staining. Arrows showed minicore changes.

Muscle biopsy was performed in patients 1, 2, 5, and 6. Most (patients 2, 5, and 6) indicated chronic myopathic changes with fiber size variation, mild atrophic and regenerative fibers, internalized nuclei, and a mild increase in endomysial connective tissue (see Figure 2). NADH staining showed that minicores present small areas of reduced oxidative enzyme reactivity. No significant changes in patient 1 were observed, and the lesions were minimal.

#### Genetic Analysis

**FIGURE 3:**
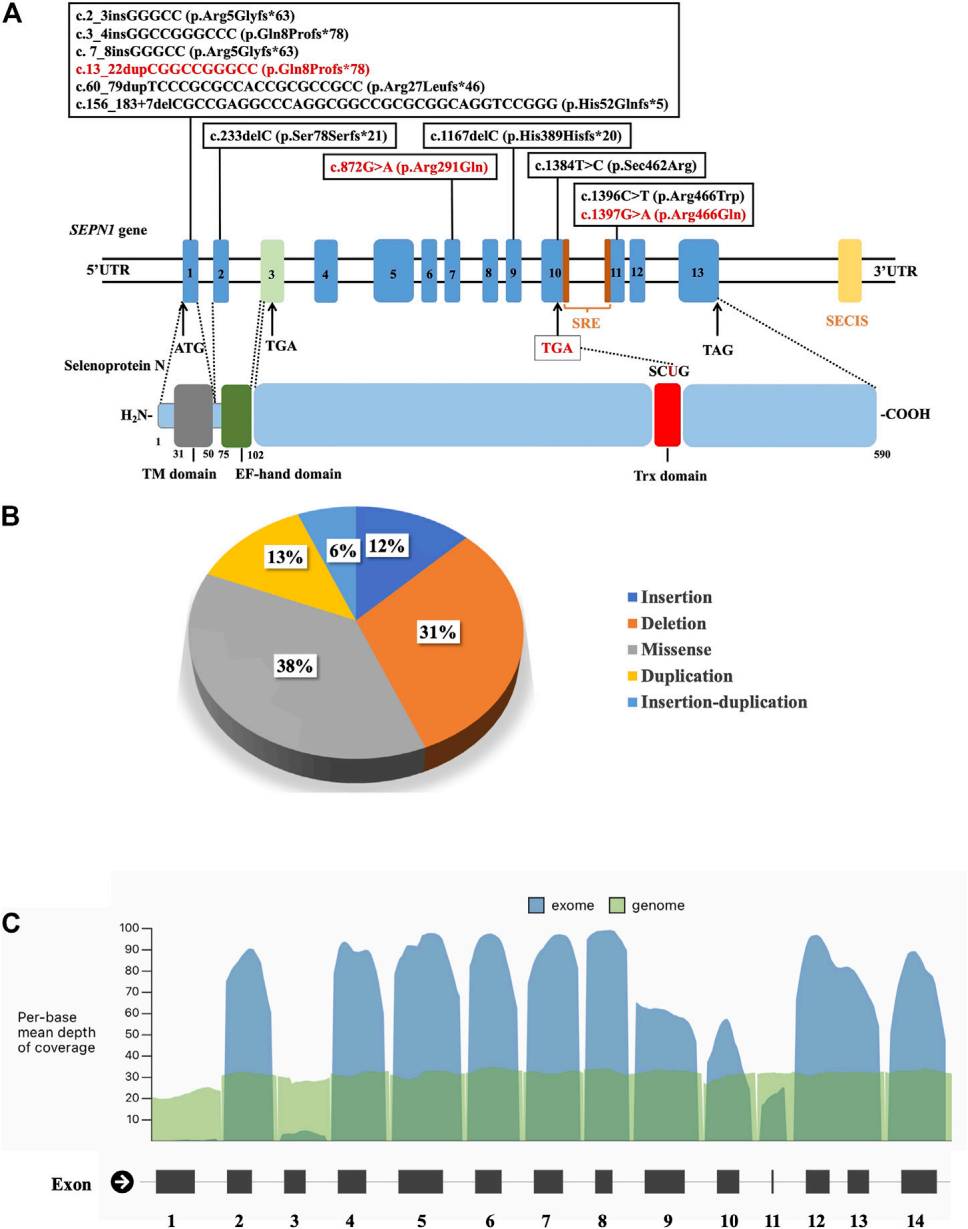
**(A)** Schematic localization of the variants identified in the *SEPN1* gene. ATG: initiation codon. EF-hand domain: Ca^2+^-interacting region. SECIS: selenocysteine insertion sequence. SRE: selenocysteine redefinition element. TAG, TGA: termination codon. TM domain: transmembrane domain. Trx domain: thioredoxin domain, encompassing SCUG motif. UTR: untranslated region. **(B)** The types of *SEPN1* variants. **(C)** Mean depth of coverage in *SEPN*1 exons and genome by next-generation sequencing (https://gnomad.broadinstitute.org).

Genomic sequencing revealed 16 compound heterozygous variants (13 unreported, 81.3%) of the *SEPN1* gene (Figure 3; Table 3), and all tested parents of probands were healthy heterozygous carriers. We observed a cluster of variants in exons 1 (7/16, 43.8%), 10 (2/16, 12.5%), and 11 (3/16, 18.8%) (Figure 3A). Exon 1, which included the high GC sequence, harbored the largest number of mutations and was the most frequent variable sequence in our cohort. The types of *SEPN1* mutations mainly included missense (6/16, 37.5%), deletion (5/16, 31.3%), insertion (2/16, 12.5%), duplication (2/16, 12.5%), and insertion–duplication (1/16, 6.2%) (Figure 3B).

**TABLE 3:** Genetic analysis of 8 RSMD1 patients.

Patient	Exon	Nucleotide change	Predicted amino acid change	Protein domain affected	Inheritance	Novel/reported
1	1	c.7_8insGGGCC	p.(Arg5Glyfs*63)	Transmembrane domain	Maternal	Novel
	9	c.1167delC	p.(His389Hisfs*20)	Non-cytoplasmic domain	Paternal	Novel
2	2	c.233delC	p.(Ser78Serfs*21)	Ca^2+^-interacting region (EF-hand domain)	Paternal	Novel
	10	c.1384T>C	p.(Sec462Arg)	SCUG motif (Selenocysteine)	Maternal	Novel
3	1	c.2_3insGGGCC	p.(Arg5Glyfs*63)	Transmembrane domain	Maternal	Novel
	1	c.3_4insdupGGCCGGGCCC	p.(Gln8Profs*78)	Transmembrane domain	Paternal	Novel
4	2	c.233delC	p.(Ser78Serfs*21)	Ca^2+^-interacting region (EF-hand domain)	Paternal	Novel
	11	c.1397G>A	p.Arg466Gln	Thioredoxin domain	Maternal	Reported
5	1	c.60_79dupTCCCGCGCCACCGCGCCGCC	p.(Arg27Leufs*46)	Transmembrane domain	Maternal	Novel
	10	c.1384T>C	p.(Sec462Arg)	SCUG motif (Selenocysteine)	Paternal	Novel
6	1	c.13_22dupCGGCCGGGCC	p.Gln8Profs*78	Transmembrane domain	Maternal	Reported
	7	c.872G>A	p.Arg291Gln	Non-cytoplasmic domain	Paternal	Reported
7	1	c.156_183+7delCGCCGAGGCCCAGGCGGCCGCGCGGCAGGTCCGGG	p.(His52Glnfs*5)	Transmembrane domain	Maternal	Novel
	11	c.1396C>T	p.(Arg466Trp)	Thioredoxin domain	Paternal	Novel
8	1	c.156_183+7delCGCCGAGGCCCAGGCGGCCGCGCGGCAGGTCCGGG	p.(His52Glnfs*5)	Transmembrane domain	Maternal	Novel
	11	c.1396C>T	p.(Arg466Trp)	Thioredoxin domain	Paternal	Novel

The mutations mainly affected the transmembrane domain and thioredoxin (Trx) domain (encompassing the SCUG motif) of selenoprotein N. Ten mutations (62.5%) result in nonsense variants and are predicted to produce truncated proteins. Most missense mutations (5/6, 83.3%) mainly affect the Trx domain, including the SCUG motif (encoding the selenoprotein N putative catalytic site), a variant (c.1384T>A), and causing a loss of selenocysteine (Sec).

## Discussion

In this study, we clinically and genetically analyzed a cohort of 8 Chinese RSMD1 patients in detail to improve our understanding of the disease, extend the disease phenotypic spectrum, and facilitate diagnosis and management of the disease. In agreement with previous studies (Scoto et al., 2011), we characterized the clinical phenotype of RSMD1 patients with delayed motor milestones, slowly progressive muscle weakness, spinal rigidity, and progressive and potentially life-threatening respiratory insufficiency.

Although all our patients had a congenital onset, they fully remained ambulant with poor running and jumping, and none needed a wheelchair for an outdoor activity. This suggests that clinical severity is independent of the age at its onset. Axial weakness was generally present with an early onset but often neglected. Since the patients showed preserved limb strength and ambulation, they rarely complained of axial weakness. The muscular disease was suspected when scoliosis and respiratory insufficiency were detected at the end of the first decade.

Joint contractures were not significant in our patients, with only two cases of long finger flexors, elbow, and Achilles tendon. As reported, the contractures most commonly affected the Achilles tendons and elbow, and less frequently the long finger flexors (Scoto et al., 2011). However, in contrast to contractures, spinal rigidity was a presenting feature and a significantly progressive factor in our cohort. *SEPN1*-RM predominantly manifests as cervico-dorsal spinal rigidity that differs from the rigid spine in myopathies caused by mutations in *LMNA* (Quijano-Roy et al., 2008), *EMD* (Kubo et al., 1998), *COL6* (Bönnemann, 2011), or *RYR1* (Bharucha-Goebel et al., 2013). Our patients presented a rigid spine at a mean age of 6.8 years and developed scoliosis at a mean age of 8.8 years, earlier than the reported age of 10 years (Scoto et al., 2011).

Pulmonary function tests showed abnormal FVC values, and PSG suggested variable hypoxia and nocturnal hypoventilation in our patients. In our patients, respiratory insufficiency was found at a mean age of 8.2 years, requiring nocturnal NIV at a mean age of 9.2 years, which was earlier than the reported age of 13 years (Villar-Quiles et al., 2020). Due to obvious diaphragmatic weakness in patients with RSMD1, sleep-related respiratory disorders are likely to occur, which will eventually lead to respiratory insufficiency (Cagliani et al., 2011). In our cohort, pulmonary hypertension secondary to respiratory insufficiency improved with nocturnal NIV, and there was no clear progressive deterioration of the respiratory function with good oxygen saturation at night. FVC values are reported to be essentially stable after the 2nd decade of life following the introduction of NIV (Scoto et al., 2011). In our cohort, one patient who refused NIV died of respiratory failure. The respiratory function declines systematically from the end of the third decade, and early NIV may initially stabilize the respiratory muscle strength (Caggiano et al., 2017). Respiratory involvement might be aggravated by spinal deformities, especially scoliosis. Indeed, one study reported that one-third of patients had a spinal fusion at a mean age of 13.9 years, with good tolerance and no deterioration of motor or respiratory function after surgery (Scoto et al., 2011). Therefore, it is important to improve the prognosis by managing scoliosis and respiratory insufficiency.

Muscle MRI in our patients revealed the involvement of the gluteus maximus and adductor magnus, and the lower leg was mildly affected. The semimembranosus is reportedly the most abnormal muscle in all *SEPN1*-RM patients (Flanigan et al., 2000). However, it was present in patient 1 but not in some RSMD1 patients (Mercuri et al., 2010). A distinct radiological pattern in *SEPN1*-RM with predominant axial and cervical muscle involvement, severe wasting of sternocleidomastoid muscles, and dramatic atrophy of semimembranosus, with relative preservation of the rectus femoris, long adductor, and gracilis, has been reported (Hankiewicz et al., 2015). This information might be useful for diagnosis even in very young patients or those with mild disease. We found no clear correlation between histopathological findings and clinical features. Muscle biopsy indicated chronic myopathic changes and minicores, as previously described (Scoto et al., 2011; Villar-Quiles et al., 2020), and the age and site of biopsy correlated with the histopathological variability. Muscle biopsy is recommended at the school age to promote diagnosis.

In our study, exon 1 harbored the highest number of mutations. Mutations in *SEPN1* are distributed along with the whole gene, except for exon 3. GC-rich exon 1 has been reported as a major hotspot, although it is poorly or not covered by NGS panels (see Figure 3C). Hence, mutations in exon 1 of *SEPN1* are easily missed by NGS, and NGS data should be re-analyzed, or the detection of exon 1 using Sanger sequencing should be performed. Moreover, the first *SEPN1* copy number variation (c.(872+1_873-1)(1602+1_1603-1)del), a large out-of-frame deletion affecting exons 7 to 12, was identified (Villar-Quiles et al., 2020). Mutations in the *SEPN1* 3′ UTR selenocysteine insertion sequence (SECIS), an untranslated cis element necessary for selenocysteine integration, were also found (Villar-Quiles et al., 2020). All these findings highlight the need to search for *SEPN1* copy number variations and mutations in SECIS, particularly when only one *SEPN1* pathogenic variant has been detected.

Most *SEPN1* mutations in our cohort were null mutations producing truncated proteins prone to nonsense-mediated decay (NMD). As reported, all mutations located around or in the sequence encoding the potential catalytic site (SCUG motif) were missense. The codon UGA encoding selenocysteine is located in exon 10 (c. 1384_1386); UGA is usually a termination codon, which causes coding termination, but selenoprotein N is unique. The SECIS located in the 3′UTR of the mRNA and the selenocysteine redefinition element (SRE) downstream of the UGA jointly mediate the selenocysteine insertion into the selenoprotein N polypeptide chain (Moghadaszadeh et al., 2013). Mutations in the codon UGA encoding selenocysteine, SECIS, and SRE can reduce the insertion efficiency of selenocysteine, reduce the level of mRNA, and ultimately affect the synthesis and expression of selenoprotein N (Maiti et al., 2009). We also detected mutations in the EF-hand domain-encoding sequence. We did not observe a clear genotype–phenotype correlation. Biallelic null mutations may be associated with higher disease severity, which is the only genotype–phenotype correlation that has been proposed (Villar-Quiles et al., 2020).

Selenoprotein N, an endoplasmic reticulum glycoprotein, is a member of an enzyme family containing one selenium atom that binds covalently to form selenocysteine in the catalytic region (Castets et al., 2012). Selenoprotein N participates in redox reactions (Moghadaszadeh et al., 2013) and calcium homeostasis (Lescure et al., 2009), protects cells from the oxidative stress (Arbogast and Ferreiro, 2010), and plays a role in early embryonic development, cell proliferation, and regeneration (Castets et al., 2009). Excessive oxidative damage induces dysfunction and degradation of muscle fibers, which is considered a primary pathophysiological mechanism of *SEPN1*-RM (Varone et al., 2019). Selenoprotein N is required for calcium transients between the ER–mitochondria, thereby controlling mitochondrial bioenergetics. The mutation of *SEPN1* leads to impaired ER–mitochondria contacts and decreased ATP levels in muscle cells (Filipe et al., 2021). The antioxidant N-acetylcysteine has been identified as an effective protective agent against oxidative stress-induced cell death (Arbogast and Ferreiro, 2010) and should be considered in future clinical trials. Multidisciplinary management should be provided to patients, including physical rehabilitation and nursing, cardiovascular and respiratory management, nutritional support, orthopedic surgery, and psychological care. We recommend yearly follow-up, including respiratory and cardiac evaluations and spine surveillance. Regular pulmonary function tests and PSG are recommended for all patients, and NIV should be used as soon as nocturnal hypoventilation or respiratory failure is detected.

In summary, our study expands the spectrum of clinical and genetic findings in RSMD1 and provides valuable genetic counseling for the families. The findings will contribute to improving diagnosis, management, and standards of care, and provide useful information on future natural history studies and clinical trials.

## Data Availability

All datasets generated for this study are included in the article/supplementary material.

## References

[B1] Arbogast S. Beuvin M. Fraysse B. Zhou H. Muntoni F. Ferreiro A. (2009). Oxidative Stress inSEPN1-Related Myopathy: From Pathophysiology to Treatment. Ann. Neurol. 65, 677–686. 10.1002/ana.21644 19557870

[B2] Arbogast S. Ferreiro A. (2010). Selenoproteins and protection against Oxidative Stress: Selenoprotein N as a Novel Player at the Crossroads of Redox Signaling and Calcium Homeostasis. Antioxid. Redox Signaling 12, 893–904. 10.1089/ars.2009.2890 19769461

[B3] Bharucha-Goebel D. X. Santi M. Medne L. Zukosky K. Dastgir J. Shieh P. B. (2013). Severe Congenital RYR1-Associated Myopathy: the Expanding Clinicopathologic and Genetic Spectrum. Neurology 80, 1584–1589. 10.1212/WNL.0b013e3182900380 23553484 PMC3662324

[B4] Bönnemann C. G. (2011). The Collagen VI-related Myopathies. Handb Clin. Neurol. 101, 81–96. 10.1016/B978-0-08-045031-5.00005-0 21496625 PMC5207779

[B5] Caggiano S. Khirani S. Dabaj I. Cavassa E. Amaddeo A. Arroyo J. O. (2017). Diaphragmatic Dysfunction in SEPN1-Related Myopathy. Neuromuscul. Disord. 27, 747–755. 10.1016/j.nmd.2017.04.010 28606403

[B6] Cagliani R. Fruguglietti M. E. Berardinelli A. D'Angelo M. G. Prelle A. Riva S. (2011). New Molecular Findings in Congenital Myopathies Due to Selenoprotein N Gene Mutations. J. Neurol. Sci. 300, 107–113. 10.1016/j.jns.2010.09.011 20937510

[B7] Castets P. Lescure A. Guicheney P. Allamand V. (2012). Selenoprotein N in Skeletal Muscle: from Diseases to Function. J. Mol. Med. 90, 1095–1107. 10.1007/s00109-012-0896-x 22527882

[B8] Castets P. Maugenre S. Gartioux C. Rederstorff M. Krol A. Lescure A. (2009). Selenoprotein N Is Dynamically Expressed during Mouse Development and Detected Early in Muscle Precursors. BMC Dev. Biol. 9, 46. 10.1186/1471-213X-9-46 19698141 PMC2739516

[B9] Chernorudskiy A. Varone E. Colombo S. F. Fumagalli S. Cagnotto A. Cattaneo A. (2020). Selenoprotein N Is an Endoplasmic Reticulum Calcium Sensor that Links Luminal Calcium Levels to a Redox Activity. Proc. Natl. Acad. Sci. USA 117 (35), 21288–21298. 10.1073/pnas.2003847117 32817544 PMC7474598

[B10] Filipe A. Chernorudskiy A. Arbogast S. Varone E. Villar-Quiles R.-N. Pozzer D. (2021). Defective Endoplasmic Reticulum-Mitochondria Contacts and Bioenergetics in SEPN1-Related Myopathy. Cell Death Differ 28, 123–138. 10.1038/s41418-020-0587-z 32661288 PMC7853070

[B11] Flanigan K. M. Kerr L. Bromberg M. B. Leonard C. Tsuruda J. Zhang P. (2000). Congenital Muscular Dystrophy with Rigid Spine Syndrome: a Clinical, Pathological, Radiological, and Genetic Study. Ann. Neurol. 47, 152–161. 10.1002/1531-8249(200002)47:2<152::aid-ana4>3.0.co;2-u 10665485

[B12] Hankiewicz K. Carlier R. Y. Lazaro L. Linzoain J. Barnerias C. Gómez-Andrés D. (2015). Whole-body Muscle Magnetic Resonance Imaging inSEPN1-Related Myopathy Shows a Homogeneous and Recognizable Pattern. Muscle Nerve 52, 728–735. 10.1002/mus.24634 25808192

[B13] Koul R. Al-Yarubi S. Al-Kindy H. Al-Futaisi A. Al-Thihli K. Chacko P. A. (2014). Rigid Spinal Muscular Dystrophy and Rigid Spine Syndrome. J. Child. Neurol. 29, 1436–1440. 10.1177/0883073813479173 23481446

[B14] Kubo S. Tsukahara T. Takemitsu M. Yoon K. B. Utsumi H. Nonaka I. (1998). Presence of Emerinopathy in Cases of Rigid Spine Syndrome. Neuromuscul. Disord. 8, 502–507. 10.1016/s0960-8966(98)00069-8 9829281

[B15] Lescure A. Rederstorff M. Krol A. Guicheney P. Allamand V. (2009). Selenoprotein Function and Muscle Disease. Biochim. Biophys. Acta (Bba) - Gen. Subjects 1790, 1569–1574. 10.1016/j.bbagen.2009.03.002 19285112

[B16] Maiti B. Arbogast S. Allamand V. r. Moyle M. W. Anderson C. B. Richard P. (2009). A Mutation in theSEPN1selenocysteine Redefinition Element (SRE) Reduces Selenocysteine Incorporation and Leads toSEPN1-Related Myopathy. Hum. Mutat. 30, 411–416. 10.1002/humu.20879 19067361 PMC2909032

[B17] Mercuri E. Clements E. Offiah A. Pichiecchio A. Vasco G. Bianco F. (2010). Muscle Magnetic Resonance Imaging Involvement in Muscular Dystrophies with Rigidity of the Spine. Ann. Neurol. 67, 201–208. 10.1002/ana.21846 20225280

[B18] Moghadaszadeh B. Rider B. E. Lawlor M. W. Childers M. K. Grange R. W. Gupta K. (2013). Selenoprotein N Deficiency in Mice Is Associated with Abnormal Lung Development. FASEB j. 27, 1585–1599. 10.1096/fj.12-212688 23325319 PMC3606527

[B19] Pozzer D. Varone E. Chernorudskiy A. Schiarea S. Missiroli S. Giorgi C. (2019). A Maladaptive ER Stress Response Triggers Dysfunction in Highly Active Muscles of Mice with SELENON Loss. Redox Biol. 20, 354–366. 10.1016/j.redox.2018.10.017 30391828 PMC6223234

[B20] Quijano-Roy S. Mbieleu B. Bönnemann C. G. Jeannet P.-Y. Colomer J. Clarke N. F. (2008). De novoLMNAmutations Cause a New Form of Congenital Muscular Dystrophy. Ann. Neurol. 64, 177–186. 10.1002/ana.21417 18551513

[B21] Richards S. Aziz N. Bale S. Bick D. Das S. Gastier-Foster J. (2015). Standards and Guidelines for the Interpretation of Sequence Variants: a Joint Consensus Recommendation of the American College of Medical Genetics and Genomics and the Association for Molecular Pathology. Genet. Med. 17, 405–424. 10.1038/gim.2015.30 25741868 PMC4544753

[B22] Scoto M. Cirak S. Mein R. Feng L. Manzur A. Y. Robb S. (2011). SEPN1-related Myopathies: Clinical Course in a Large Cohort of Patients. Neurology 76, 2073–2078. 10.1212/WNL.0b013e31821f467c 21670436

[B23] Varone E. Pozzer D. Di Modica S. Chernorudskiy A. Nogara L. Baraldo M. (2019). SELENON (SEPN1) Protects Skeletal Muscle from Saturated Fatty Acid-Induced ER Stress and Insulin Resistance. Redox Biol. 24, 101176. 10.1016/j.redox.2019.101176 30921636 PMC6438913

[B24] Villar-Quiles R. N. von der Hagen M. Métay C. Gonzalez V. Donkervoort S. Bertini E. (2020). The Clinical, Histologic, and Genotypic Spectrum of SEPN1-Related Myopathy. Neurology 95, e1512–e1527. 10.1212/WNL.0000000000010327 32796131 PMC7713742

[B25] Zhu S. Y. Liu L. L. Huang Y. Q. Li X. W. Talukder M. Dai X. Y. (2021). In Silico analysis of Selenoprotein N (Gallus gallus): Absence of EF-Hand Motif and the Role of CUGS-helix Domain in Antioxidant protection. Metallomics 13, mfab004. 10.1093/mtomcs/mfab004 33693771

